# Zona incerta modulation of the inferior olive and the pontine nuclei

**DOI:** 10.1162/netn_a_00350

**Published:** 2024-04-01

**Authors:** Ramakrishnan Bhuvanasundaram, Samantha Washburn, Joanna Krzyspiak, Kamran Khodakhah

**Affiliations:** Dominick P. Purpura Department of Neuroscience, Albert Einstein College of Medicine, New York, NY, USA

**Keywords:** Zona incerta, Inferior olive, Cerebellum, In vivo electrophysiology, Optogenetics, In vitro electrophysiology

## Abstract

The zona incerta (ZI) is a subthalamic structure that has been implicated in locomotion, fear, and anxiety. Recently interest has grown in its therapeutic efficacy in deep brain stimulation in movement disorders. This efficacy might be due to the ZI’s functional projections to the other brain regions. Notwithstanding some evidence of anatomical connections between the ZI and the inferior olive (IO) and the pontine nuclei (PN), how the ZI modulates the neuronal activity in these regions remains to be determined. We first tested this by monitoring responses of single neurons in the PN and IO to optogenetic activation of channelrhodopsin-expressing ZI axons in wild-type mice, using an in vivo awake preparation. Stimulation of short, single pulses and trains of stimuli at 20 Hz elicited rapid responses in the majority of recorded cells in the PN and IO. Furthermore, the excitatory response of PN neurons scaled with the strength of ZI activation. Next, we used in vitro electrophysiology to study synaptic transmission at ZI-IO synapses. Optogenetic activation of ZI axons evoked a strong excitatory postsynaptic response in IO neurons, which remained robust with repeated stimulation at 20 Hz. Overall, our results demonstrate a functional connection within ZI-PN and ZI-IO pathways.

## Author Summary

We have demonstrated that zona incerta (ZI) neurons are capable of communicating effectively with the neurons in the two major types of precerebellar nuclei: the pontine nucleus and the inferior olive. The two circuits revealed by our research might play a critical role in tremor-related movement disorders by engaging the cerebellar network. Furthermore, these circuits could explain why deep brain stimulation of the ZI is effective for patients with tremor-related motor disorders.

## INTRODUCTION

The zona incerta (ZI) is a diencephalic region located ventral to the thalamus. It interconnects with various structures including the cerebral cortex (Barthó et al., 2007; Lin et al., 1990), superior colliculus (May & Basso, 2018; May et al., 1997), spinal cord (Bittencourt & Elias, 1998; Shaw & Mitrofanis, 2001), basal ganglia (Heise & Mitrofanis, 2004), and cerebellum (Aumann et al., 1994, 1996; Mitrofanis & deFonseka, 2001). While the majority of cells in the ZI are GABAergic (Kolmac & Mitrofanis, 1999; Mitrofanis et al., 2004), there are also populations of glutamatergic (Heise & Mitrofanis, 2004; Kolmac & Mitrofanis, 1999), dopaminergic (Sharma et al., 2018; Sita et al., 2003), nitric oxide synthase (NOS^+^) (Mitrofanis et al., 2004), and melanin-concentrating hormone (MCH^+^) neurons (Bittencourt & Elias, 1998; Sita et al., 2003). At least four sectors of the ZI have been described across species: rostral (rZI), dorsal (dZI), ventral (vZI), and caudal (cZI) (Mitrofanis et al., 2004; Romanowski et al., 1985). The ventromedial ZI, adjacent to the subthalamic nucleus (STN), is considered the main part of the subthalamic locomotor region (Milner & Mogenson, 1988; Waller, 1940), which is thought to be involved in initiating locomotion, based on several lines of evidence from various studies (Milner & Mogenson, 1988; Mori et al., 1989; Waller, 1940; Whelan, 1996). Electrical stimulation of the subthalamic locomotor region has been shown to induce greater locomotor activity than stimulation of the STN in freely moving rats (Milner & Mogenson, 1988; Supko et al., 1991). Recently, the ZI has been shown to modulate other behaviors, such as fear generalization and retrieval, regulation of novelty seeking in monkeys by a subregion of the ZI (Ogasawara et al., 2022), defense behavior via GABAergic subpopulations (Chou et al., 2018; Venkataraman et al., 2019; Zhou et al., 2018), and differential encoding and modulation of anxiety by subpopulations of ZI neurons (Li et al., 2021).

Given its prominent role in the generation of locomotion, the ZI has been studied in the context of movement disorders. Abnormal oscillations with burst firing have been reported in the ZI in patients with Parkinson’s disease (PD), a movement disorder characterized by degeneration of dopamine neurons in the substantia nigra pars compacta and, among other symptoms, difficulty initiating voluntary movements (Merello et al., 2006). The neurons in the ZI became hyperactive in 6-OHDA-lesioned rodent models of PD (Périer et al., 2000), and inhibiting the cZI activity ameliorated the motor deficits (Li et al., 2022). The motor performance of 6-OHDA-induced PD mice was significantly improved by the activation of GABAergic neurons in the ZI, and repeated activation of GABAergic neurons increased the dopamine content in the striatum (Chen et al., 2023). In humans, a lesion involving the ZI and the subthalamic nucleus (STN) produced a greater clinical benefit in PD patients than those who had undergone an STN lesion alone (Patel et al., 2003). Recently, the cZI in particular has been shown to be an effective target for deep brain stimulation (DBS) in patients with tremor-related movement disorders (Blomstedt et al., 2012, 2018; Fytagoridis et al., 2012; Plaha et al., 2008, 2011), such as essential tremor (Awad et al., 2020; Fytagoridis et al., 2012; Holslag et al., 2018; Plaha et al., 2008) and PD (Blomstedt et al., 2012, 2018; Plaha et al., 2006). A case study of patients suffering from a range of tremors (parkinsonian, Holmes, cerebellar, essential, multiple sclerosis, and dystonic tremors) found that bilateral DBS of the ZI improved all aspects of the patients’ tremors (Plaha et al., 2008).

Studies have shown that tremors are not caused by a single malfunctioning node of the brain, but a network of pathological oscillatory activity that includes the inferior olive (IO), cerebellum, thalamus, and motor cortex (Raethjen & Deuschl, 2012). The ZI is being explored as a new target in tremor treatment in part because there is a very close link between the ZI and the cerebellar network, as there are extensive interconnections between them (Aumann et al., 1994, 1996; Border & Mihailoff, 1991; Mihailoff, 1995; Mitrofanis & deFonseka, 2001). The ZI receives dense projections from the deep nuclei of the cerebellum (Aumann et al., 1994, 1996). The ZI sends both GABAergic and glutamatergic projections to the pontine nuclei (PN) (Border et al., 1986; Border & Mihailoff, 1991), which relay signals from vast regions of the cortex and various other parts of the brain to the cerebellum through their axons, called mossy fibers. Moreover, previous work shows that the ZI has connections with the IO (Ricardo, 1981), which sends its axons to the cerebellum as climbing fibers, and are known to convey error or teaching signals there. However, the details of the ZI-IO connections have not been documented.

While previous studies describe the presence of two-way anatomical connections between the ZI and the cerebellar network (Aumann et al., 1994, 1996; Border & Mihailoff, 1991; Mihailoff, 1995; Mitrofanis & deFonseka, 2001), whether these projections are functional and are able to modulate the neuronal activity within the targeted area has not been determined. In this study, we combined optogenetics, in vitro electrophysiology, and in vivo electrophysiology in awake, head-restrained mice to examine the properties of ZI inputs to the neurons in the PN and IO. We found that the ZI modulates the activity of neurons in the PN and IO with a short latency, presumably via monosynaptic inputs.

## MATERIALS AND METHODS

All the experiments were approved by the Institutional Animal Care and Use Committee (IACUC) of the Albert Einstein College of Medicine in New York. Mice were maintained on a 12-hour reversed light/dark cycle.

### Animals

Experiments were performed on C57/B6 wild-type mice of both sexes. Mice of 7–10 weeks old were used for in vivo experiments, whereas in vitro experiments were performed in sagittal slices obtained from juvenile mice of 5–6 weeks old.

### Stereotactic Surgery and Virus Injection

Mice were anesthetized with 5% isoflurane and maintained at 1–2% on stereotactic apparatus (Kopf). Mice were injected with 0.25/0.15 *μ*l of AAV2-hSyn-ChR2-YFP (UNC vector core) at a rate of 0.1 *μ*l/min using a 32-gauge syringe (Hamilton Gastight 1701), and an automated injector unit (Stoelting). The injections were performed targeting the center of the ZI (coordinates: Bregma −2.1 mm AP, ± 1.5 mm ML, −4 mm DV). At the end of surgery, mice were injected with an analgesic (flunixin, 2.5 mg/kg). The mice were given at least 2 weeks to recover from the surgical procedure. This period also optimized the expression of channelrhodopsin (ChR2).

For our experiments, we wanted to maximize viral transfection with ChR2 throughout all the sectors of the ZI. However, the location and elongated shape of these sectors made meeting this objective problematic. In our experiments, ChR2 was injected into the center of the ZI. The injection covered a substantial portion of the ventral and dorsal sectors (Supporting Information Figure S1A) and a moderate part of the rostral and caudal sectors. Cases where the ChR2 spread far beyond the ZI were excluded from our analysis.

### In Vivo Electrophysiology

Following injections, mice were implanted with a flat titanium head-restraint metal plate over the pontine nuclei using Metabond (C&B Metabond Quick Adhesive Cement System, Parkell, S380). The craniotomy was performed (coordinates: Bregma −3.7 to −4.7 mm, AP; ± 0 to 1.5 mm ML) and an optrode was lowered into the pontine nuclei for neuronal recording. Optrodes were constructed using a 100-*μ*m optical fiber (Multimode Fiber, 0.22 NA, High-OH 105 *μ*m Core, ThorLabs, FG105UCA) attached to a tungsten electrode (WPI, TM33B20, shaft diameter = 0.256 mm) using epoxy (Devcon 5 Minute Epoxy). Optrodes were coated with DiI (Invitrogen, V22889) for post hoc identification of the recording sites (Supporting Information Figure S1B, right).

Extracellular single-unit recordings were performed while stimulating optogenetically with a 447-nm laser (OEM Laser Systems). Based on the Paxinos and Franklin atlas, the following coordinates were used for PN neuronal recordings: −3.8 to −4.6 mm, AP; ± 0 to 1.2 mm ML; 4.5 mm and below DV and for IO neuronal recordings: −6.9 to −7.4 mm, AP; ± 0.3 to 0.5 mm ML; 3.9 mm and below DV. For optogenetic stimulation, 1-ms light pulses were delivered at 5-second intervals. To explore whether the cells could encode the strength of their synaptic input, different light intensities (low, medium, and high) were used. Cells that did not respond to a maximum light intensity of 8 mW were considered “no response” (NR) cells. For train stimulation, five 1-ms light pulses were delivered at 20 Hz with an interstimulus interval of 10 seconds. At least 40 trials were repeated for each cell.

Signals were amplified ×2,000 using a customized differential amplifier (1 Hz to 10 kHz, RC band-pass filter), digitized at 20 kHz (National Instruments, PCI6052E), and collected using custom-written software in LabVIEW (National Instruments). Single units were sorted using principal component analysis (Plexon Offline Sorter) and analyzed with custom-written programs in MATLAB (MathWorks). Firing rates were calculated from the peri-stimulus time histogram (PSTH) using bins of 2 ms. Based on response type the recorded cells were categorized as excitation (Ex), inhibition (In), excitation followed by inhibition (Ex + In), and NR.

Extra spikes were calculated by counting the number of spikes that occurred within 30 ms after the stimulus onset, and subtracting this from the number of spikes that would have occurred if the firing rate had stayed the same as viewed from the baseline (calculated by taking the average firing rate for 3 seconds before the stimulus onset). Excitatory response latencies were defined as the duration between the stimulus onset and the time the firing rate reached 3 standard deviations above the baseline. An inhibition response was identified when the spike count in three consecutive bins was less than 1 standard deviation of the mean baseline spike count.

### In Vitro Electrophysiology

Two weeks after the injections, mice were euthanized with isoflurane and transcardially perfused with an ice-cold NMDG solution aCSF (Ting et al., 2014) (in mM: NMDG 93, KCl 2.5, NaH_2_PO_4_ 1.2, NaHCO_3_ 30, Glucose 25, HEPES 20, Na-ascorbate 5, Na-pyruvate 3, Thiourea 2, MgSO_4_ 10, CaCl_2_ 0.5), and decapitated. The brain was quickly removed and placed in ice-cold NMDG aCSF and then secured with super glue to a metal platform and placed in a bath of NMDG aCSF at 2–4°C. Sagittal sections of 250 *μ*m were made on a vibrating microtome (Campden Instruments Model 7000smz-2). Slices were incubated in NMDG aCSF at 34°C for less than 15 minutes and then placed in standard aCSF (in mM: 125 NaCl, 2.5 KCl, 26 NaHCO3, 1.25 NaH2PO4, 1 MgCl2, 2 CaCl2, and 11 glucose) at room temperature for at least an hour. Slices were used until up to 6 hours after slicing. All solutions were continuously bubbled with 95% oxygen/5% carbon dioxide. Slices were transferred to a recording chamber and kept under continuous perfusion with oxygenated aCSF at a rate of 1.5–2 ml/min, and maintained at 32–35°C. Recording electrodes were made from filamented borosilicate glass (Sutter BF150-110-10HP), pulled to a resistance of 2.5–4 MΩ (Sutter Model P-97), and filled with internal solution containing (in mM) 70 Cs-gluconate, 10 CsF, 20 CsCl, 10 EGTA, 10 HEPES, 3 Na2ATP, and 2 QX-314. Furthermore, 2 mM of Neurobiotin Tracer (Vector Laboratories, SP-1120) was added to the internal solution. Slices were examined with a ×40 objective under oblique infrared illumination (Zeiss). Whole-cell recordings were made with a Cairn Optopatch amplifier, and cells were clamped at −60 mV unless otherwise stated. The ChR2-expressing ZI axons in the field of view were optogenetically activated through the objective using an LED (455 nm, ThorLabs M455L3). All pulses for stimuli were 1 ms in duration. The maximum stimulus intensity measured at the output of the ×40 objective did not exceed 5 mW. Whole-cell data were acquired at 10 kHz (PCI-MIO-16XE-10 or 6052E; National Instruments) using custom-written software in LabVIEW (National Instruments). Data were analyzed in LabVIEW and MATLAB (Mathworks).

### Histology

After in vivo recordings, mice were anesthetized with isoflurane and transcardially perfused with phosphate buffered solution (Fisher Scientific) followed by 4% paraformaldehyde (Acros Organics). The brain was removed and fixed overnight in 4% PFA at 4°C. The brain was cryoprotected in 30% sucrose for 30–40 hours and then frozen in cryoprotectant solution (OCT) on dry ice and stored at −80°C. Brains were sectioned at 40-*μ*m thickness using a cryostat (Leica CM3050S). Sections were stained with primary antibody against GFP (chicken, 1:1,000) (Abcam AB13970) and incubated overnight at 4°C, followed by the secondary antibody Alexa 488 (1:1,500, goat anti-chicken, Invitrogen Life Technologies A11039) along with nuclei-label DAPI (1:2,000, Hoescht 33342, Invitrogen Life Technologies H1399). The slides were viewed under a fluorescent microscope (Zeiss) at ×10–40 objectives, which was then used to capture images. Recording sites were determined by the presence of DiI fluorescence and damage indicated by the optrode tract (Supporting Information Figure S1B, right). Mice which during post hoc histological examination exhibited ChR2 expression beyond the ZI were excluded from the analysis.

After in vitro recording, slices were fixed in 4% PFA overnight at 4°C and then stained with primary antibodies against GFP, followed by secondary antibodies Alexa 647 (1:1,000, goat anti-rabbit, Invitrogen Life Technologies A32733), Streptavidin conjugated to Alexa 568 (2 *μ*g/mL, 1:50, Invitrogen Life Technologies, S11226), and Alexa 488 for examination.

### Statistics

The analyses were performed in MATLAB (MathWorks), LabVIEW (National Instruments), and Prism (GraphPad). Data are reported as mean ± *STD*, unless otherwise specified. Throughout the text, “*N*” refers to the number of animals, while “*n*” refers to the number of cells.

## RESULTS

### Activation of Zona Incerta Inputs Rapidly Alters Neuronal Activity in Pontine Nuclei

**Figure 1.**
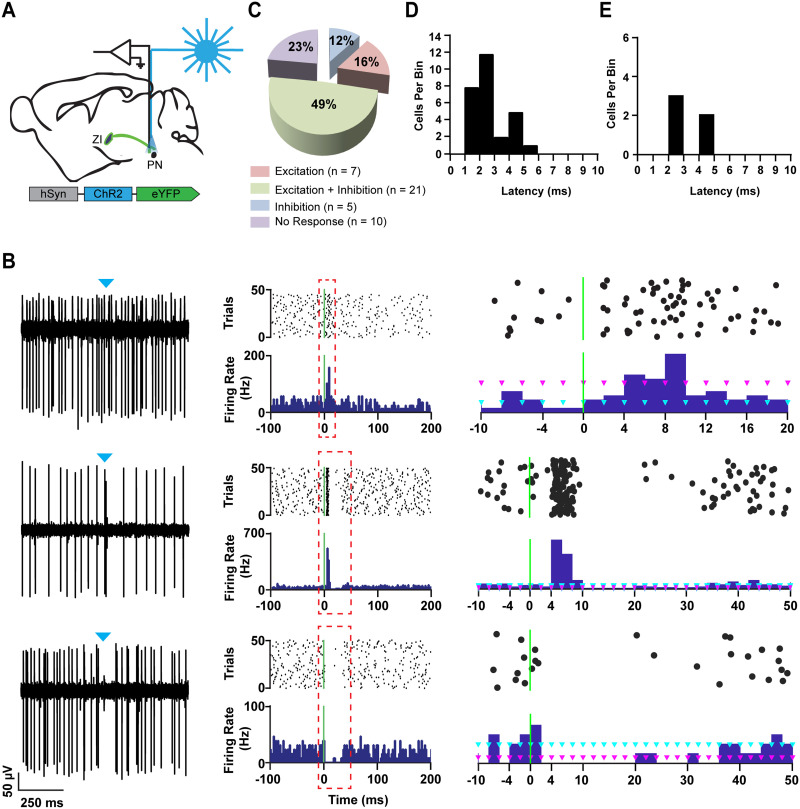
A rapid short-duration optogenetic activation of zona incerta axons reliably elicits responses in the pontine nuclei. (A) A schematic representation of the experiment, in which single units were recorded extracellularly in the pontine nuclei while activating ChR2-expressing ZI fibers. (B) Left: A representative raw trace is shown for each response type. The blue triangle represents the stimulus onset. Top - excitation; middle - excitation + inhibition; bottom - inhibition. (B) Center: A PSTH of a responsive cell with its corresponding raster plot. The green vertical line represents the stimulus onset. Top - excitation; middle - excitation + inhibition; bottom - inhibition. (B) Right: A zoomed-in representation of the dotted-box area, showing the stimulus onset and the subsequent response in a short time window. Light blue triangles indicate mean firing rate. Pink triangles delineate 3 standard deviations above the mean for excitation and 1 standard deviation below the mean for excitation + inhibition, and inhibition. (C) A pie chart with the distribution of responses. More than 75% of the recorded cells responded to stimulation with excitation (16%), excitation + inhibition (49%), and inhibition (12%); *n* = 43, *N* = 5. (D) A latency histogram of excitation and excitation + inhibition cells. (E) A latency histogram of inhibition cells.

We sought to determine whether ongoing activity of neurons in the PN can be altered under in vivo conditions by inputs from the ZI. First, the presence of ZI projections to the PN was confirmed by injecting an adeno-associated virus expressing the excitatory opsin channelrhodopsin (ChR2), and a yellow fluorescent protein (YFP) reporter (AAV2-hSyn-ChR2 (H134R)-eYFP) into the ZI of wild-type C57BL/6 mice (Supporting Information Figure S1A). Our histological results revealed the presence of ChR2-expressing ZI axons in the PN (Supporting Information Figure S1B). Using an optrode, we then recorded extracellular single-unit activity in the PN of awake, head-restrained mice while we stimulated the ChR2-expressing ZI axons with 1-ms pulses of blue light (Figure 1A). Activating the ChR2-expressing axons near the recording site rapidly altered firing in 77% of examined PN neurons (number of cells *n* = 43; number of animals *N* = 5) (Figure 1B). Three response types were observed: excitation (Ex, *n* = 7; *N* = 4), excitation followed by inhibition (Ex + In, *n* = 21; *N* = 4), and inhibition (In, *n* = 5; *N* = 3) (Figures 1B and 1C). Unresponsive cells were categorized as “no response” (*n* = 10; *N* = 5) (Figure 1C). The mean latency of excitation, including the peak of excitation followed by inhibition, was 2.39 ± 0.2 ms (Figure 1D), while the mean latency of inhibition was 2.8 ± 0.49 ms (Figure 1E). Together, our results confirm that ZI fiber activation could rapidly elicit responses in the PN neurons.

### The Neurons of Pontine Nuclei Follow Repeated Activation of Zona Incerta Inputs

**Figure 2.**
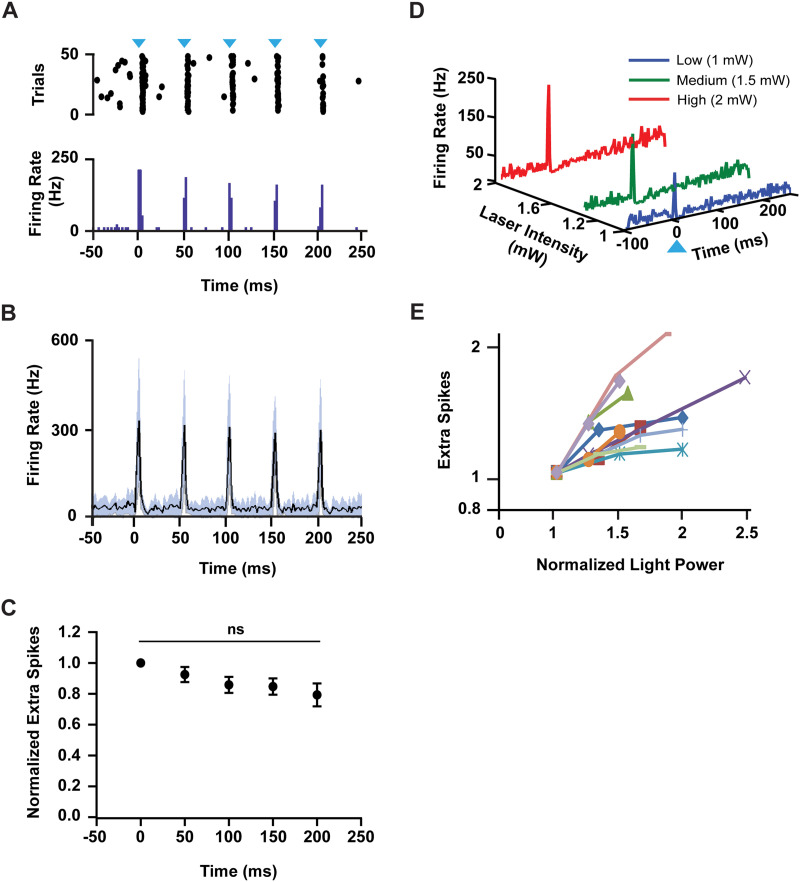
Neurons in the pontine nuclei can follow trains of input from the zona incerta, and the response of excited cells scales with graded inputs. (A) A PSTH of a representative cell with its corresponding raster plot that followed a 20-Hz train of stimulation of ZI axons. The blue triangle represents the stimulus onset. (B) The mean (black line) ± *STD* (grey area) of all the neurons that followed the train of stimulation. (C) A plot showing the calculation of the average extra spikes of all the cells evoked by each pulse within the train. Note: The response of each pulse is normalized to the response of the first pulse (mean ± *SEM*); *n* = 7, *N* = 4; *F* (2.162, 10.81) = 0.8862, *P* = 0.4478. (D) A 3D plot of a representative cell, highlighting the graded excitation response to increasing light stimulation intensities. The blue triangle represents the stimulus onset. (E) Extra spikes of all the responsive cells, plotted against light power. Note: The responses were normalized to 1-mW light power; *n* = 10, *N* = 4.

ZI projection neurons are spontaneously active and can fire action potentials at ~20 Hz (Merello et al., 2006). Therefore, we investigated whether the ZI-PN synapses could follow trains of input at this frequency. We monitored PN neuron activity in response to five 1-ms light pulses delivered as a 20-Hz train of stimuli (Figure 2A) in awake, head-restrained mice. We found that the firing rate of PN neurons (*n* = 7; *N* = 4) increased in response to each pulse in the train (Figure 2B) and exhibited little sign of depression. The average decrease in response amplitude from the first pulse to the second was 8.64 ± 2.69%, while from the first pulse to the last was 21.22 ± 3.15% (Figure 2C).

### Excitatory Response of Pontine Nuclei Neurons Is Graded to the Strength of Activation of Zona Incerta Inputs

Whether the PN neurons can encode the strength of ZI inputs was tested by activating ZI axons with varying light intensities while monitoring the evoked excitatory responses in the PN in awake, head-restrained mice. We observed that the amplitude of the excitatory response increased in proportion to the stimulus intensity and tested this with at least three stimulation powers (Figure 2D). The graded responses to increased stimulation were observed in several examined PN neurons (*n* = 10; *N* = 4) (Figure 2E). The average number of extra spikes for the three conditions are as follows: low 0.92 ± 0.16; medium 1.14 ± 0.19; and high 1.33 ± 0.23. This suggests that the ZI projections to the PN may, in principle, encode information in a graded manner.

### Activation of Zona Incerta Inputs Evokes a Strong Response in the Inferior Olive

**Figure 3.**
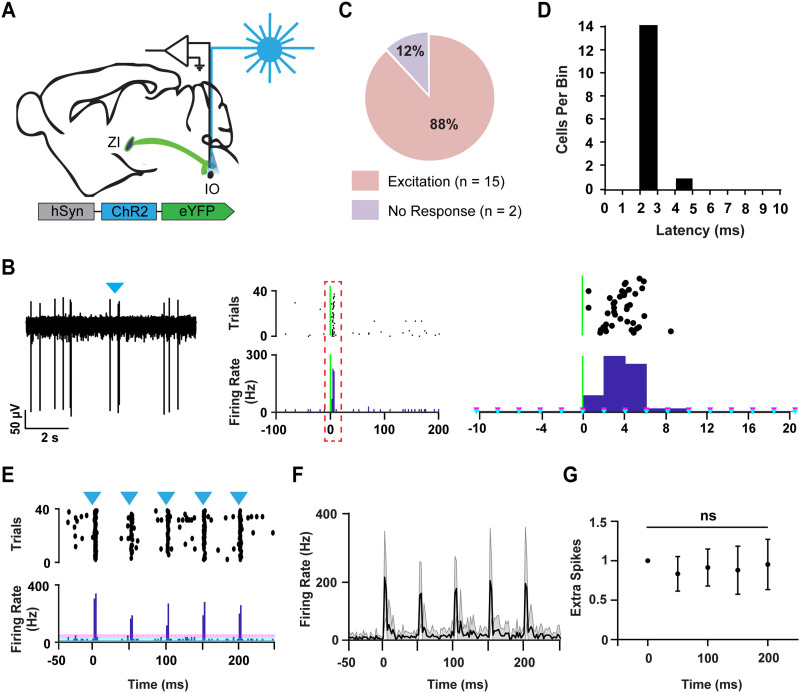
Neurons in the inferior olive respond to single pulses and trains of light stimulation of zona incerta axons under in vivo condition. (A) A schematic representation of the experiment, where single units were recorded in the inferior olive while activating ZI fibers. (B) Left: An example raw trace is shown. The blue triangle represents the stimulus onset. (B) Center: A PSTH of the responsive cell with its corresponding raster plot. The green vertical line represents the stimulus onset. (B) Right: A zoomed-in representation of the dotted-box area, showing the stimulus onset and the subsequent response in a short time window. Light blue triangles indicate mean firing rate. Pink triangles delineate 3 standard deviations above the mean. (C) A pie chart highlighting the response profiles of the neurons recorded. Almost 90% of the recorded cells exhibited excitation; *n* = 17, *N* = 4. (D) A latency histogram of neurons that responded to ZI inputs with excitation. (E) An example cell that followed a 20-Hz train of stimulation. The blue triangle represents the stimulus onset. Light blue triangles indicate mean firing rate. Pink triangles delineate 3 standard deviations above the mean. (F) The mean and STD of all the neurons that responded to the 20-Hz train stimulation; *N* = 7, *N* = 4. (G) Average extra spikes in response to each pulse within the train were calculated for all the cells that responded to the stimulation. The responses of the additional pulses are normalized to the first pulse (mean ± *SEM*); *n* = 7, *N* = 4.

**Figure 4.**
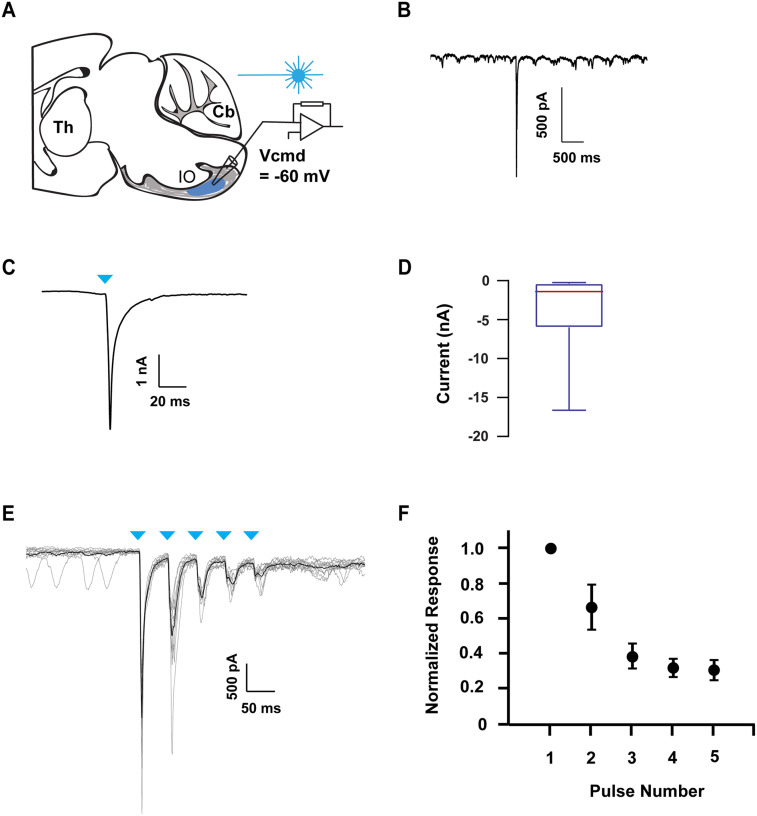
The zona incerta directly and efficiently modulates inferior olive neurons. (A) An experimental schematic. Neurons in the IO were recorded in whole-cell voltage clamp, and zona incerta axons were stimulated through the field of view with single 1-ms pulses or five 1-ms pulses at 20 Hz. (B) An example sweep. Inferior olive neurons are known to exhibit oscillatory activity due to gap junction coupling, which can be seen in the sweep. (C) An average of 10 responses of an example IO neuron to 1-ms pulses of light. (D) A boxplot showing the distribution (median, igr, range) of responses of IO neurons to the stimulation of zona incerta inputs; *n* = 14, *N* = 7. (E) Raw traces of a representative IO neuron that responded to the stimulation of zona incerta axons with five 1-ms pulses delivered at 20 Hz. The blue triangle represents the stimulus onset. (F) A summary of all the cells that responded to a 20-Hz train of stimulation. Note: the response of each pulse is normalized to the response of the first pulse; *n* = 8, *N* = 7.

Similarly to the PN, we examined whether ZI projections to the IO were capable of driving the activity of IO neurons under in vivo conditions. We histologically verified the presence of ChR2-expressing ZI axons in the IO in wild-type mice in which the ChR2 tagged viral construct was injected into the ZI (Supporting Information Figure S2B1–B3). As in PN single-pulse experiments, extracellular single-unit activity was recorded in the IO while activating ZI axons with 1-ms pulses of blue light (Figure 3A). The activation of ZI axons near the recording site rapidly altered firing in 88% of examined IO neurons (*n* = 17; *N* = 4). However unlike PN, we only observed excitation responses in the IO (Ex, *n* = 15; *N* = 4) (Figures 3B and 3C). The mean latency of excitation was 2.13 ± 0.1 ms (Figure 3D). Although the placement of the optrode within the IO following each in vivo experiment (Supporting Information Figure S2B1–B3) was histologically verified, to unequivocally establish whether ZI projections can drive synaptic activity in the IO, we performed patch-clamp recordings in acutely prepared sagittal slices of the IO from juvenile mice injected with AAV2-hSyn-ChR2-YFP in the ZI (Figure 4A). In the whole-cell voltage clamp configuration, excitatory postsynaptic currents (EPSCs) were evoked by optogenetic activation of ZI axons with 1-ms light pulses in the IO (Figure 4B and 4C) in the majority of recorded cells (*n* = 14; *N* = 7) (Figure 4D). The results from both in vivo and in vitro indicate that the ZI projections can reliably drive activity in the IO.

### Inferior Olive Neurons Follow Repeated Activation of Zona Incerta Inputs

Next, we examined whether IO neurons could follow a train of 20-Hz input from ZI neurons, as they are known to fire spontaneously at approximately this frequency (Merello et al., 2006). Under in vivo conditions, we monitored IO neuron activity in response to five 1-ms light pulses delivered as a 20-Hz train of stimuli (Figure 3E) in awake, head-restrained mice. We found that the firing rate of IO neurons increased accordingly in response to each pulse in the train (Figures 3F and 3G). Similar to in vivo, we found that trains of ZI stimulation with each pulse of light evoked EPSC responses in the IO neurons in vitro (*n* = 8; *N* = 7) (Figure 4E). The amplitude of the response was depressed 33 ± 11% from the first to the second pulse, and 67 ± 4.6% from the first to the last pulse (Figure 4F). These results suggest that the IO neurons can follow a train of input from the ZI.

## DISCUSSION

In this study, we have demonstrated that the inputs from the ZI strongly modulated the activity of the neurons in the pontine nuclei and in the IO, the two main precerebellar nuclei, which are known to send their axonal projections to the cerebellum as mossy and climbing fibers, respectively. These functional projections may thus play a key role in cerebellar physiology.

The ZI contains both glutamatergic and GABAergic projection neurons and sends both of the projections to the neurons in the pontine nuclei (Border et al., 1986; Border & Mihailoff, 1991). Glutamatergic cells, while concentrated in the dorsal ZI, are also present in all other sectors (Kolmac & Mitrofanis, 1999), whereas GABAergic cells are most distinct in the ventral zona incerta but are also present in other sectors (Kolmac & Mitrofanis, 1999; Mitrofanis et al., 2004). There are two GABAergic subpopulations: a subpopulation expressing parvalbumin^+^ (PV^+^), which is mainly found in the ventral ZI (Kolmac & Mitrofanis, 1999; Mitrofanis et al., 2004), and one expressing somatostatin^+^ (SOM^+^), which is mainly found in the rostral sector and also sparsely in the ventral and dorsal sectors (Kolmac & Mitrofanis, 1999). We observed that the majority of the recorded cells in the PN and the IO responded to rapid ZI stimulation, indicating that the ZI sends strong functional projections to these areas. Various response types were found in the PN, of which the dominant one was excitation followed by inhibition, suggesting that a single neuron might receive inputs from both the excitatory and the inhibitory neurons in the ZI.

One of the limitations in this study is that our ChR2 injection only partially covered the ZI region, precluding us from providing a detailed connectivity pattern within the PN. Additional research using various specific transgenic mice should yield a detailed characterization of the ZI connectivity with the PN neurons.

Earlier anatomical study has shown that the ZI has connections with the IO (Ricardo, 1981), although the details of these connections have not been documented. As a result, we characterized this connection with both in vivo and in vitro electrophysiology. In our in vivo preparation, there was only a limited ability to access the IO with an optrode. Therefore, in addition to in vivo electrophysiology, we utilized in vitro electrophysiology to test our hypothesis that the input from the ZI modulates the activity of the neurons in the IO. To our knowledge, this is the first study that has documented the properties of the ZI projections to the IO. In the in vivo experiments, activating ZI inputs only evoked excitation responses in the IO, unlike in the PN. Similarly, in our in vitro experiments, we observed strong EPSCs in olivary neurons while stimulating ZI fibers. Much to our surprise, we only found excitatory responses in both in vivo and in vitro preparations in IO neurons that responded to the optical stimulation, which suggests that, despite the large population of GABAergic projection neurons in the ZI, the IO might only receive functional inputs from the excitatory neurons of the ZI. However, it is worth noting that our sample size is small, and due to technical complications in the in vivo experiment, likely did not cover the entire IO. We cannot therefore exclude the possibility that the IO receives a GABAergic input from the ZI that can modulate the neuronal activity.

### Functional Implications of the Zona Incerta-Pontine Nuclei Pathway

The ZI has been shown to be vulnerable in PD patients and PD animal models (Li et al., 2022; Merello et al., 2006; Périer et al., 2000). Heterogeneous neuronal signals ranging from tonic to burst firing at 4 and 20 Hz, abnormal oscillations, and reduced extracellular background activity in the ZI have been reported in PD patients (Merello et al., 2006). Moreover, the neurons in the ZI were hyperactive in PD rats (Périer et al., 2000), and a study in PD mice showed that glutamatergic neurons in the cZI regulated parkinsonian motor symptoms. In these mice the cZI neurons were hyperactive, and inhibiting their activity ameliorated the motor deficits (Li et al., 2022). The area of these aberrant neurons was mainly situated dorsal to the STN, which is implicated in locomotion (Li et al., 2022; Milner & Mogenson, 1988; Waller, 1940). Moreover, the neurons in the neighboring STN are known to become aberrant in PD patients (Levy et al., 2000) and PD animal models (Lintas et al., 2012). The cerebellum is also abnormally affected by PD (for a review, see Wu & Hallett, 2013). Several pathological changes have been reported to occur in the cerebellum of parkinsonian patients (Benninger et al., 2009; Rolland et al., 2007). In line with earlier suggestions (Bostan et al., 2010; Sutton et al., 2014), we propose that the abnormal patterns of neuronal firing might be transmitted from the ZI and the STN to the cerebellar network through the PN. This is further supported by the recent discovery in mice of the existence of an anatomical route from the STN to the cerebellar cortex via the PN (Bhuvanasundaram et al., 2022), which had previously been suggested by a primate study (Bostan et al., 2010). Together, the motor sector of the ZI and the motor division of the STN are part of the subthalamic locomotor region, which is assumed to be involved in the initiation and execution of locomotion (Milner & Mogenson, 1988; Waller, 1940) (for a review, see Whelan, 1996). Therefore, the transmitting signals may encode an aberrant signal that contributes to the impairment of locomotion in PD. The transmitting information might also cause the cerebellum to play a compensatory role under PD conditions, as the cerebellum has been shown to be hyperactive in PD patients (Festini et al., 2015; Liu et al., 2013; Yu et al., 2007).

In 6-OHDA rats, the striatal extracellular glutamate level was increased, while ZI lesions reduced the raised glutamate levels back to normal, leading to improved symmetry in forepaw use (Walker et al., 2010). In 6-OHDA-induced PD mice, the motor performance was improved by the activation of GABAergic neurons in the ZI (Chen et al., 2023). A lesion involving the ZI yielded a greater clinical benefit in patients who had undergone subthalamotomy for PD (Patel et al., 2003). Several studies have reported that DBS in the cZI was effective in alleviating parkinsonian syndromes, especially tremor (Blomstedt et al., 2012, 2018; Plaha et al., 2006, 2008). Therefore, we hypothesize that the effect of DBS in the ZI might be propagated to the cerebellar network via the ZI-PN pathway. The effect might be propagated in parallel through ZI inputs to other motor structures that are either directly or indirectly influenced by the disease condition (Awad et al., 2020). Future studies focusing on exploring the hypothesis mentioned above are likely to be of benefit to clinical applications.

### Functional Implications of the ZI-IO Pathway

Recently, ZI-DBS has been shown to be an effective and safe therapy for essential tremor (Fytagoridis et al., 2012; Plaha et al., 2008). Bilateral ZI-DBS is mainly effective in suppressing the postural and action components of essential tremor (ET) (Plaha et al., 2011) and is shown to be a treatment for long-term suppression (Fytagoridis et al., 2012). The ZI-DBS effect has also been demonstrated to be superior to VIM-DBS (Holslag et al., 2018). DBS in cZI is also shown to be a safe target from a cognitive perspective in the treatment of ET (Philipson et al., 2019). Previous studies focusing on cZI-DBS for ET have suggested that DBS of the cZI is likely to suppress tremor symptoms by acting on oscillations in the IO and various other structures via projections from the ZI neurons (Plaha et al., 2008, 2009). A modeling study produced a prediction that the IO neurons can trigger neural oscillations that are propagated to a more extensive network and indicated that these neurons may be involved in tremor oscillations via the dentato-rubro-olivary pathway (Zhang & Santaniello, 2019). We have found, for the first time, the presence of a strong functional connection driven by an excitatory input. Therefore, the functional projection through the ZI-IO pathway might play a role in the impacts of ZI-DBS, which may exert additional effects on the olivocerebellar loop in ET patients. Further studies should help to delineate the proposed mechanism.

## CONCLUSION

Overall, our results demonstrate that the ZI establishes a strong functional connection with the pontine nuclei and the IO. Although our experiments do not unequivocally show monosynaptic connections in the ZI-IO and ZI-PN pathways, the short latency responses suggest that these connections are likely monosynaptic. We propose that these projections play a role in alleviating tremor-related motor symptoms achieved by ZI-DBS in PD and ET patients.

## ACKNOWLEDGMENTS

We thank the members of the Khodakhah lab.

## SUPPORTING INFORMATION

Supporting information for this article is available at https://doi.org/10.1162/netn_a_00350.

## AUTHOR CONTRIBUTIONS

Ramakrishnan Bhuvanasundaram: Conceptualization; Data curation; Formal analysis; Investigation; Methodology; Visualization; Writing – original draft; Writing – review & editing. Samantha Washburn: Formal analysis; Methodology; Visualization; Writing – review & editing. Joanna Krzyspiak: Formal analysis; Writing – review & editing. Kamran Khodakhah: Funding acquisition; Supervision; Writing – review & editing.

## FUNDING INFORMATION

Kamran Khodakhah, National Institute of Neurological Disorders and Stroke (https://dx.doi.org/10.13039/100000065), Award ID: R01NS105470.

## Supplementary Material



## TECHNICAL TERMS

Subthalamic locomotor region (SLR): The subthalamic brain region, including the zona incerta, that is thought to be involved in initiating locomotion.
Parkinson’s disease (PD): Neurodegenerative disorder caused by dopamine depletion in the substantia nigra pars compacta and characterized by tremor and slow movement.
Deep brain stimulation (DBS): The stimulation of deep brain regions using chronically implanted electrodes and a pulse generator.
Essential tremor (ET): A progressive neurological disorder characterized by involuntary and rhythmic shaking of certain body parts.
Mossy fibers (MF): Fiber bundles that relay signals from cortical regions and various other parts of the brain to the cerebellum.
Climbing fibers (CF): The axons of the inferior olive (IO) that are known to convey error or teaching signals to the cerebellum.
Excitatory postsynaptic current (EPSC): The flow of ions that makes the postsynaptic neuron more likely to fire an action potential.
