# Supplementary material for: Zona incerta modulation of the inferior olive and the pontine nuclei
Source: Netw Neurosci. 2024 Apr 1;8(1):260–74. doi: 10.1162/netn_a_00350 (PMC10927296; doi:10.1162/netn_a_00350)
Supplement: Supplementary file 1 [file netn-8-1-260-s001.pdf]

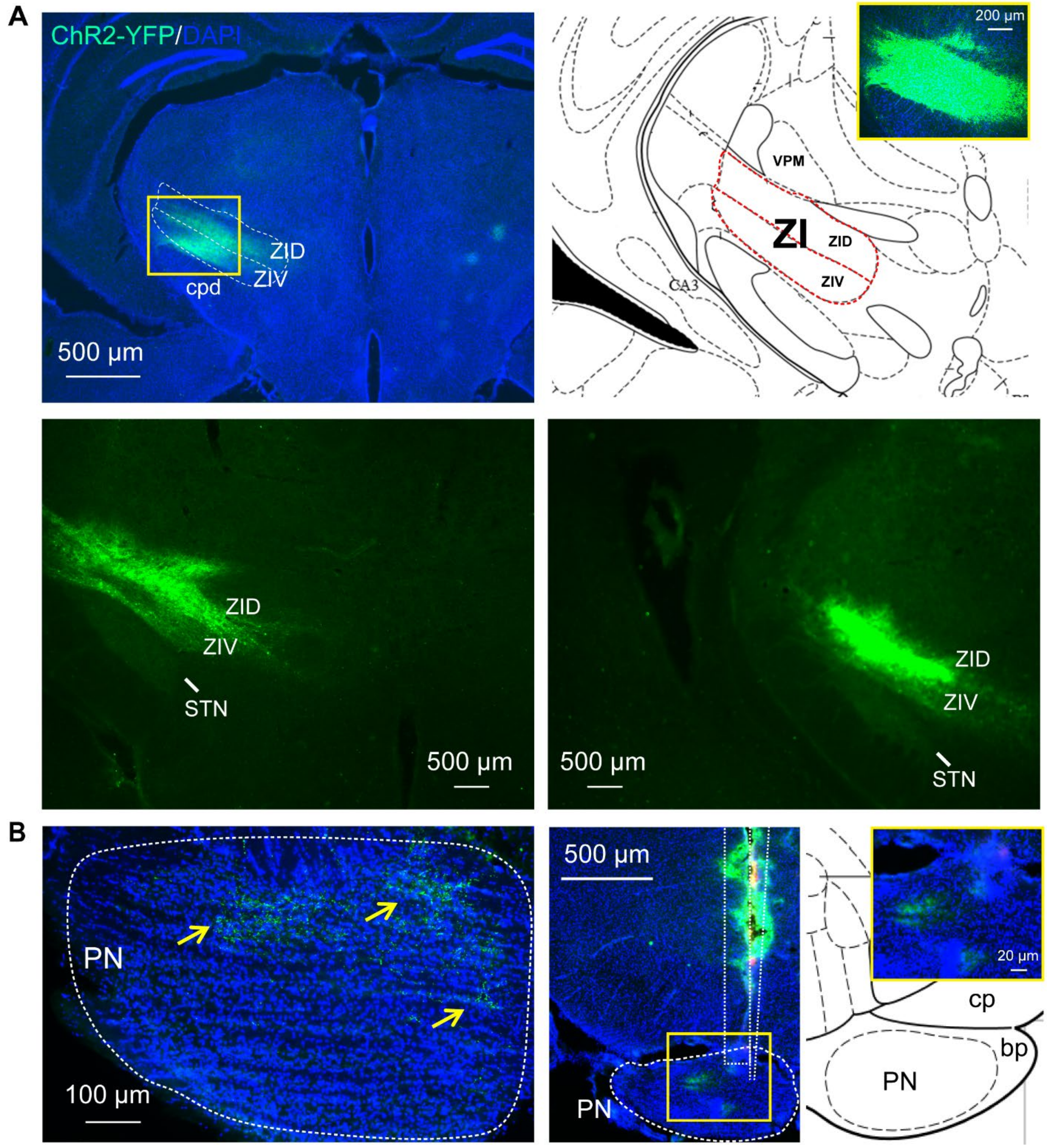

Supplementary Figure 1: Post-hoc confirmation of the expression of channelrhodopsin in the targeted zona incerta and the recording sites in the pontine nuclei

(A) Left: AAV2-hSyn-ChR2-YFP was injected into the left zona incerta. Note that the spread of the virus stayed within the dorsal and ventral zona incerta.

(A) Right: A zoomed-in representation of the boxed area in the image on the left, along with a diagram of the corresponding brain region, adapted from the Paxinos & Franklin Atlas.

(A) Bottom: Two example sections from a different mouse, highlighting the presence of ChR2 expression in the zona incerta. However, no expression was found in the neighboring areas, in particular the STN. We show GFP images without DAPI because the border between the zona incerta and the STN was clearer, and these two regions are clearly distinguishable.

(B) Left: ChR2-expressing ZI axonal fibers are present in the pontine nuclei; these fibers are indicated by yellow arrows.

(B) Right: The presence of DiI in the pontine nuclei, highlighting the optrode tract, along with a zoomed-in representation of the boxed area, in which the ChR2-expressing zona incerta fibers are visible, and a diagram of the corresponding brain region, adapted from the Paxinos & Franklin Atlas.

ZID - dorsal zona incerta, ZIV - ventral zona incerta, STN - subthalamic nucleus, PN - pontine nuclei, cpd - cerebral peduncle.

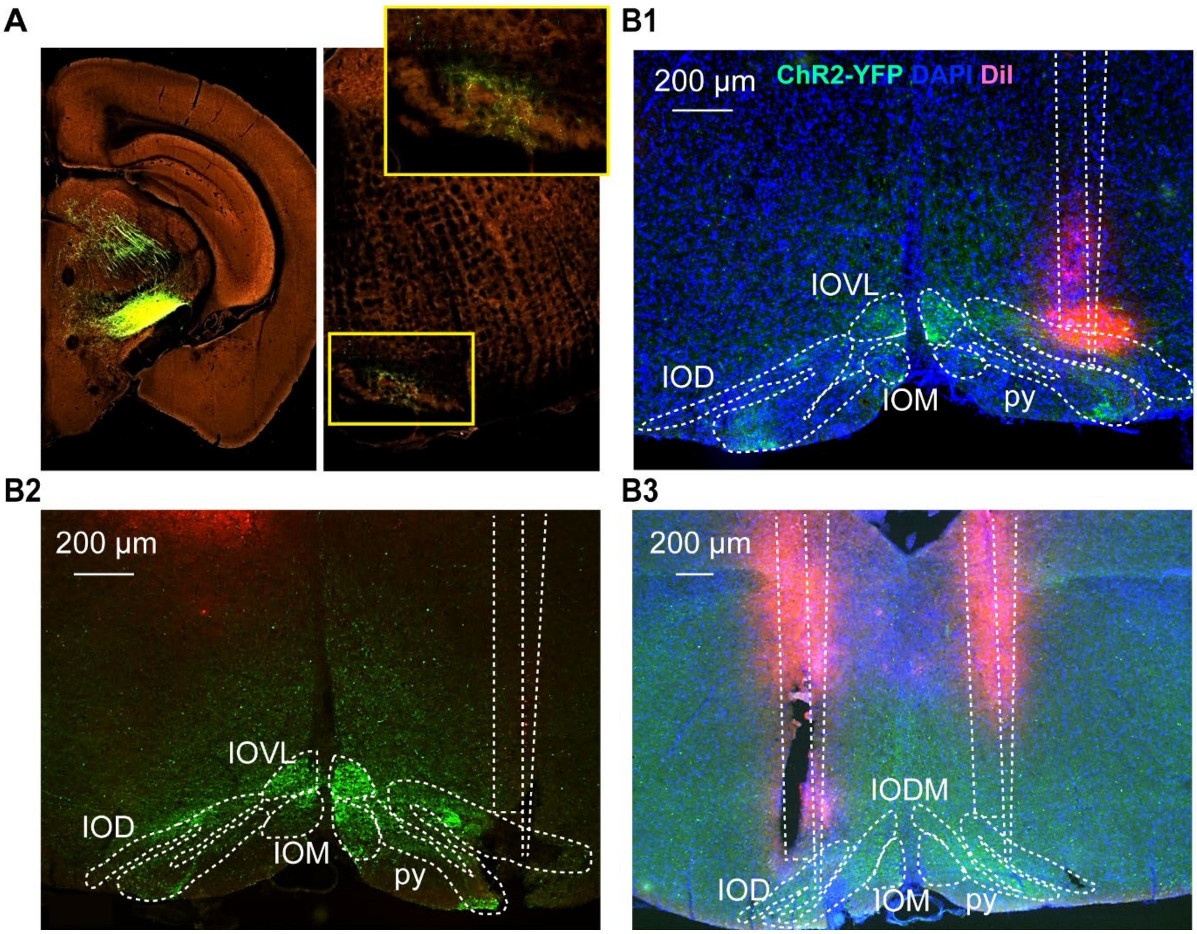

Supplementary Figure 2: The presence of channelrhodopsin-expressing zona incerta axonal fibers and optrode placements in the inferior olive

(A) Left: A section taken from the Allen Brain Atlas (ID – 162018879), showing the injected ZI area, which contains a dense GFP expression (left) and the ipsilateral inferior olive (right). A zoomed-in representation of the boxed area shows the GFP-expressing axonal fibers of the ZI in the inferior olive.

(B1, B2, & B3): A DiI track caused by optrode penetration (dotted line) confirming the recorded area within the IO. One example section from each mouse is shown. N=3.

IOD – inferior olive dorsal, IOVL – inferior olive ventrolateral, IOM – inferior olive medial, IODM – inferior olive dorsomedial.
